# Involvement of stakeholders in determining health priorities of adolescents in rural South Africa

**DOI:** 10.3402/gha.v9.29162

**Published:** 2016-03-15

**Authors:** Rhian Twine, Kathleen Kahn, Alexandra Scholtz, Shane A. Norris

**Affiliations:** 1 MRC/Wits Rural Public Health and Health Transitions Research Unit (Agincourt), School of Public Health, Faculty of Health Sciences, University of the Witwatersrand, Johannesburg, South Africa; 2 Umeå Centre for Global Health Research, Epidemiology and Global Health Unit, Department of Public Health and Clinical Medicine, Umeå University, Umeå, Sweden; 3 INDEPTH Network, Accra, Ghana; 4 F. Hoffmann-La Roche AG, Basel, Switzerland; 5 MRC/Wits Developmental Pathways for Health Research Unit, Department of Paediatrics, Faculty of Health Sciences, University of the Witwatersrand, Johannesburg, South Africa

**Keywords:** stakeholder involvement, stakeholder, adolescents, health priorities, Delphi

## Abstract

**Background:**

When developing intervention research, it is important to explore issues from the community perspective. Interventions that promote adolescent health in South Africa are urgently needed, and Project Ntshembo (‘hope’) aims to improve the health of young women and their offspring in the Agincourt sub-district of rural northeast South Africa, actively using stakeholder involvement throughout the research process.

**Objective:**

This study aimed to determine adolescent health priorities according to key stakeholders, to align stakeholder and researcher priorities, and to form a stakeholder forum, which would be active throughout the intervention.

**Design:**

Thirty-two stakeholders were purposefully identified as community members interested in the health of adolescents. An adapted Delphi incorporating face-to-face discussions, as well as participatory visualisation, was used in a series of three workshops. Consensus was determined through non-parametric analysis.

**Results:**

Stakeholders and researchers agreed that peer pressure and lack of information, or having information but not acting on it, were the root causes of adolescent health problems. Pregnancy, HIV, school dropout, alcohol and drug abuse, not accessing health services, and unhealthy lifestyle (leading to obesity) were identified as priority adolescent health issues. A diagram was developed showing how these eight priorities relate to one another, which was useful in the development of the intervention. A stakeholder forum was founded, comprising 12 of the stakeholders involved in the stakeholder involvement process.

**Conclusions:**

The process brought researchers and stakeholders to consensus on the most important health issues facing adolescents, and a stakeholder forum was developed within which to address the issues. Stakeholder involvement as part of a research engagement strategy can be of mutual benefit to the researchers and the community in which the research is taking place.

## Paper context

To inform an intervention addressing health problems facing adolescents in rural South Africa, we involved lay and professional stakeholders. Three workshops brought researchers and stakeholders to consensus, using the Delphi method and participatory visualisation, on the most important issues facing adolescents, and a stakeholder forum was formed. It was agreed that intervention needs to focus on behavioural change and improving health literacy. As the intervention goes ahead, there will be continuous involvement of the stakeholder forum.

## Background

Although there is a growing literature on stakeholder involvement in health research (1, 2), there is still a need for literature describing the *how* and effects of such involvement (3). This paper describes one step in a process of stakeholder involvement, using a variety of adapted methods that aimed to achieve consensus between researchers and professional and lay stakeholders regarding the health priorities of adolescents in a rural area of South Africa.

Almost one in five persons in the world is an adolescent, totalling 1.2 billion people ages 10–19 years globally. The state of their health is important for their lives now and in the future, and young women's health will impact on the next generation (4). There is increasing evidence that risk profiles developed during adolescence – including poor eating habits and lack of physical exercise, resulting in obesity – can lead to chronic non-communicable diseases later in life (5). Sub-Saharan African populations have the fastest growing proportion of adolescents, coupled with the worst regional adolescent health profile (6).

Among South African adolescents, the percentage of overweight female adolescents increased from 24.3% in 2002 to 29.0% in 2008, while female obesity rose from 5.0 to 7.5% over the same period (7). In rural South Africa, levels of female adolescent overweight and obesity reached a prevalence of 25% at 18 years of age (8), while high levels of stunting persisted in childhood (9). In South Africa, 27.3% of women under the age of 20 years nationally reported already having a child, and there was an HIV prevalence of 5.5% in females aged 15–19 years (10). These results indicate that South Africa, both urban and rural, is well into the epidemiologic transition, confronting increasing risk of non-communicable disease while simultaneously dealing with infectious diseases, especially the HIV epidemic (11).

Given the importance of adolescent health for future adult health, adolescence may offer a unique window of opportunity to intervene and positively impact on individuals’ health trajectories into adulthood (5). Consequently, we are developing a community-based intervention to optimise the health of young women in South Africa. Project Ntshembo, meaning ‘hope’ in the local Tsonga language, plans to create a continuum of care-seeking and self-care behaviour from pre-pregnancy through pregnancy, childbirth, and infancy.

There is a growing awareness of the importance of taking into account perspectives and experiences of people other than researchers in determining the relevance of research and uptake of its results (12). Participating populations and their local service providers understand the relevance of research to their communities (13) and can be involved as stakeholders in research projects, assisting in ensuring the respect, empowerment, and protection of populations making up research participants (14).

An essential and early stage in the development of the Project Ntshembo intervention is to understand health priorities from the perspective of the community where the intervention will be delivered and evaluated. We therefore defined a series of stakeholder involvement activities for the duration of the planned project, as follows.

*Project start-up*: Involving stakeholders at this stage should help shape the research agenda. The aim is to discuss issues sufficiently so that all stakeholders perceive the issue as important and to gain consensus about health priorities for adolescents. This ensures that stakeholders are on board in appreciating the pertinence of the research.

*Preliminary findings*: Sharing preliminary findings with stakeholders not only increases awareness but can tease out issues, helping to shape, refine, and ensure that later stages of intervention development are targeted appropriately.

*Project progression*: Through ongoing involvement during research progress, stakeholders can assist with problem-solving, for example by contributing ideas to improve cohort retention or adherence to intervention strategies.

*Project end*: If stakeholder involvement has been effective, such stakeholders could become, or engage with, policy ‘champions’ to act on research findings – potentially enabling the outputs to be used more widely and have greater impact.

*Beyond the project*: If the intervention proves effective or lessons learnt are important, the dynamic should shift to policy champions and stakeholders using the researchers as stakeholders while they endeavour to implement policy changes (15).

The aim of this paper is to describe stakeholder involvement in the start-up phase of Project Ntshembo, specifically to gain consensus on the priority health needs of adolescents in rural areas of South Africa, using an adapted Delphi technique; to align stakeholder- and researcher-identified priority health needs of adolescents using participatory visualisation; and to establish a stakeholder forum.

## Methods

### Study setting

This study took place in the Agincourt health and socio-demographic surveillance system (HDSS) site, which has been run by the Medical Research Council/Wits University Rural Public Health and Health Transitions Research Unit since 1992. The site covers 420 km^2^ in the Agincourt sub-district of rural north-east South Africa, Mpumalanga Province, 500 km from Johannesburg, with the Kruger National Park on its eastern border. The area is representative of rural areas of South Africa in that it fits into one of two definitions of *rural* appearing in the *Comprehensive Rural Development Framework Version 1 July 2009* – settlements in the former apartheid homelands, with no major economic base apart from migrant labour and remittances, typified by poverty and underdevelopment and where traditional authorities operate a land tenure system (16). In 2013, the population was 111,500 people in 18,500 households in 31 villages. Although some 30% of the sub-district population comprises former Mozambican refugees, over 80% of these people are now South African permanent residents or citizens. From 2004, people holding permanent residence have been able to access all government services, and this ability has improved the livelihoods of the former Mozambican refugees and enabled their successful integration (17). Since 1994, the area has seen an increase in infrastructure development: prepaid electricity is now available in all villages, gravel roads are being tarred, and a programme of improving water provision is underway, albeit slowly. The quality of education is poor, although every village has at least one primary school and most have a high school. There are two health centres and six clinics within the Agincourt HDSS site, with three district hospitals 25–60 km away. A process of community engagement in research has evolved in this site over the years, with a dedicated LINC office (learning, information dissemination, and networking with the community) established in 1994. This office is responsible for community engagement and liaises closely with civic and traditional village leadership, as well as local service providers (18), resulting in relationships of mutual trust and respect.

### Study design

**Fig. 1:**
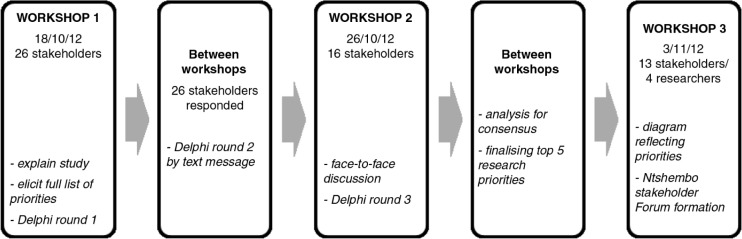
Overview of study design.

In the first two of a series of three workshops (Fig. 1), we used an adapted Delphi technique and face-to-face discussions during workshops to determine stakeholder priorities on health needs of adolescents. The Delphi technique is a group facilitation technique that was developed as a research method to deal with opinions, not facts (19). The method seeks to obtain consensus on the opinions of ‘experts’ (referred to as *stakeholders* in this paper) through a number of sequential voting rounds, using anonymously completed questionnaires with the responses from each questionnaire fed back in summarised form to the experts. Additional rounds of voting are conducted until consensus is reached. Usually, the Delphi is conducted with groups of experts with similar levels of expertise, who do not know who the other participants are and who never actually meet. The most common method of conducting the Delphi is via post, email, or mobile phone (20). We adapted the Delphi in that some stakeholders knew each other, there were both lay and professional experts in the stakeholder group, and we held workshops where stakeholders came together for face-to-face discussion on results and for anonymous voting. Given the rural context, the LINC office considered the face-to-face format most suitable, since relying on post or email was not feasible. Although all stakeholders were literate, levels of literacy varied and discussion during the consensus process ensured that all stakeholders had the same understanding of the aims of the study. We used text messaging for voting between workshops. All activities were conducted in English.

### Participants

There is no absolute consensus on what defines a community in any process of stakeholder involvement in research (13, 21). However, for work conducted by and with the LINC office, the community is defined as those individuals living in the Agincourt HDSS site who could be participants in the research or be affected by the research activities, as well as groups, organisations, and service providers who could be involved with, interested in, or affected by the research activities in the site.

Given that health is inextricably linked to a broad range of social determinants, inputs from various perspectives are needed to cover the range of health priorities of rural adolescents. The LINC office purposefully identified key individuals from the community interested in the health of adolescents who were already part of the LINC office network as stakeholders (research participants and their local service providers) for this study. A group of 32 diverse community members were invited to participate, including lay and professional stakeholders representing the following sectors: South African Department of Health (District), public health clinics, Department of Education (District), high school educators, local and district government, youth service providers, community leadership, African National Council (ANC) Youth League (this population predominantly supports the ANC, which was at the time the only political party with an active youth league in the area), MRC/Wits-Agincourt Unit Community Advisory Group, male and female adolescents, and parents.

### Data collection

#### Determining stakeholder adolescent health priorities

The first workshop aimed to explain the study, describe the adapted Delphi technique, elicit a list of adolescent health priorities from the stakeholders, and conduct the first round of voting. Facilitators collected questionnaires listing adolescent health priorities that the stakeholders had previously completed and generated a full list of all issues mentioned in these questionnaires. Stakeholders were then asked to vote on what they individually thought were the top 10 health priorities from the full list in a secret ballot, ranking them from most to least important. Results were then collated and the top five adolescent health priorities were identified.

The top five priorities generated during Round 1 were sent by text message to each participant before the second workshop with detailed instructions to 1) validate the consolidated list of issues and 2) rank the priorities from the list. A ranked list was thus generated as Round 2.

As the final step in the Delphi process, we had to test consensus. Stakeholders attended a second workshop, where they were given the results from Round 2, divided into groups for discussion, and then asked to present their discussion points in a plenary. Finally, they were asked to individually re-rank the priorities in a secret ballot.

Data analysis determined that consensus was reached on the top five ranked adolescent health priorities based on the final vote conducted during the second workshop.

#### Aligning stakeholder and researcher priorities

**Fig. 2:**
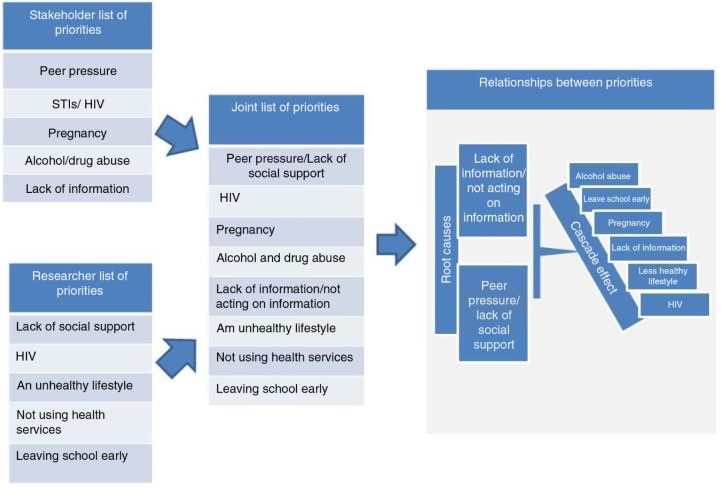
Diagram showing the relationships between stakeholder and researcher adolescent health priorities.

Once stakeholder consensus on the top five adolescent health priorities was reached, a final workshop was conducted to engage stakeholders around the results of formative research relevant to Project Ntshembo that had recently been carried out in the site. Formative research was a critical first step to inform all aspects of intervention design (22). It focused on adolescent health and access to services (23); overweight and obesity (8); community beliefs and practices around adolescent health, pregnancy, delivery, and infant feeding (24); access to food and dietary choice (25); and attitudes and perceptions of young women regarding physical activity (26). This final workshop aimed to align stakeholder views with scientific evidence. Participatory visualisation (27) was used to generate a diagram (Fig. 2) showing the relationships between the two sets of priorities. In this technique, topics (in this case the two lists of priorities) are written onto separate pieces of paper, which can be stuck to a wall and moved around by any group member until their position shows their relationship to each other, to the agreement of all group members. Participatory visualisation ensures equal participation, produces a large number of ideas, and provides a sense of closure that is often not found in less structured group methods. Stakeholders and researchers were randomly allocated into four smaller groups to brainstorm on both sets of priorities. Each group reported back to the plenary. Further discussion was facilitated, and all group members were encouraged to move the priorities appearing on the papers around on the wall until agreement was reached. Finally, a diagram was developed showing the links between the main issues affecting adolescents in a rural area of South Africa. Field notes recording the discussions were taken, because the content of the discussions added depth to the description of the relationships between priorities.

#### Forming a stakeholder forum

Additionally, during the final workshop suitable candidates were identified to become members of the Ntshembo Stakeholder Forum. Stakeholders first discussed criteria for inclusion in the stakeholder forum and then nominated and voted for forum members. They also identified organisations in the area that already provided, or had the potential to provide, services to adolescents, with which the researchers could work during the intervention.

### Ethics approval

Prior to study commencement, ethical approval was obtained from University of the Witwatersrand Human Research Ethics Committee (Medical) (certificate number M2120661), and written permission was obtained from the Mpumalanga Provincial Government Department of Education and Department of Health and Social Development, for their officials to be involved. Signed informed consent was obtained from all study participants.

### Data analysis

When using Delphi, it is important to know when consensus has been reached so as to make the right decision about when to stop the voting process. We determined consensus through the use of non-parametric statistics, as proposed by Schmidt (19). Non-parametric analysis is statistical analysis used where the data do not need to fit a normal distribution and need to be ranked. The mean rank for each priority was calculated, taking into account the number of times it was voted for and its ranking, and consensus was assessed using the Kendall coefficient, *W*, in non-parametric analysis. A coefficient of 0.1 and under shows weak agreement, whereas 0.7 and above indicates strong agreement and that consensus has been reached (28). Values of 0.9 to 1.0 indicate unusually strong agreement.

## Results

### Adolescent health priorities identified by stakeholders

Fifty issues faced by adolescents were originally listed by stakeholders – ranging through health issues (e.g. HIV, pregnancy, obesity), social issues (e.g. peer pressure, uninvolved parents), moral issues (e.g. lack of spiritual education, cultural norms), community issues (e.g. lack of water, violence, poverty), and difficulties inherent in being an adolescent (e.g. hormones, attitudes). In the first round of voting, a list of 10 issues was prioritised, with five issues receiving 50% or more of the votes as shown in Table 1.

**Table 1:** Results of Rounds 1, 2, and 3 of the Delphi voting

Round 1		Round 2		Round 3	
		
Priority	% of stakeholders who voted for this issue	Priority	% of stakeholders who voted for this issue	Priority	Final ranked list
Peer pressure	65	Peer pressure	52	Peer pressure	1.03
Pregnancy	64	Pregnancy	40	STIs/HIV	2.66
Alcohol/drug abuse	51	STDs/HIV	44	Pregnancy	3.09
Lack of information	39	Lack of information	44	Alcohol/drug abuse	4
STIs/HIV	37	Alcohol/drug abuse	24	Lack of information	5
Crime	36				
Obesity	31				
Lack of respect/discipline	28				
Poverty	27				
Hormones	25				

STIs, sexually transmitted infections.

In the 7 days between the first and second workshops, these five issues were sent by text message to the stakeholders in random order and they were asked to confirm that these were the most important issues and rank them from most important to least important. All stakeholders responded confirming that these five were the most important issues. The final ranking, shown in Table 1, followed voting at the end of the second workshop. At this stage, we tested consensus and because Kendall's coefficient (*W*) was 0.718 (strong consensus), we were able to accept this ranked list as the final result.

Peer pressure remained as the top priority throughout all voting rounds. Sexually transmitted infections (STIs) and HIV moved up in each consecutive round and finally ranked second. Pregnancy moved from second to third most important during the process, and alcohol/drug abuse and lack of information were finally ranked fourth and fifth.

### Aligning stakeholder- and researcher-identified priority health needs of adolescents

At the final workshop, the researchers presented the top five adolescent health needs they had identified from previous formative research in the site, both published and unpublished. They were as follows, in no particular order: leaving school early, not using health services, leading an unhealthy lifestyle, lack of social support, and HIV.

Participatory visualisation brought the similar, but not identical, priorities of the researchers and stakeholders into a useful diagram (Fig. 2).

During deliberations, it was agreed by all participants (stakeholders and researchers) that two priorities could be joined between the stakeholders’ and the researchers’ lists, making a final list of eight priorities. More obviously, *HIV* from the stakeholders’ list and *HIV/STIs* from the researchers’ list were grouped as one priority. Less obviously, *lack of social support* from the researchers’ list and *peer pressure* from the stakeholders’ list were grouped as one. Stakeholders and researchers felt that behaviours encouraged through peer pressure could be discouraged if appropriate social support was available. As one stakeholder noted:

Because of being poor, it is not only peers who force you, but the situation can lead to the family forcing you to do things.

During the plenary, stakeholders and researchers unanimously agreed that *peer pressure* and *lack of information or having information but not acting on it* were the root causes of all the other priorities, hence their position at the top of the diagram. In the words of one stakeholder:

Lack of information (or not acting on information) starts it all. We need to take responsibility; otherwise you fall prey to peer pressure, and alcohol abuse, pregnancy, HIV follow.

Further discussion focused on how all of the priorities were linked, creating a ‘crossover, cascade’ effect. For example, lack of information and peer pressure were identified as clear links to possible pregnancy, which could cause adolescents to leave school early. Conversely, leaving school early could lead to lack of information as well as pregnancy.

Interestingly, there was not much discussion about HIV as a health priority among adolescents, although it was listed in the final eight priorities. Some discussion centred on a perception that young people seem to be more afraid of becoming pregnant than of contracting HIV and other STIs. Most of the discussion about HIV focused on adolescents not acting on information and not accessing health services.

People know about HIV and how to prevent it – but they either ignore the information, or decide not to access the available health services.

This is another example of how the group felt that the priorities crossed over and caused cascades of problems.

Although obesity did not feature as one of the top eight topics, the group discussions identified that having an unhealthy lifestyle can be linked directly to obesity. Discussions around obesity centred on youth being lazy and watching too much television. This applied especially to young women, who no longer need to walk so far to fetch water and do not play football or other sports. Again, discussion linked this to lack of information, with one stakeholder noting that

Not acting on information is linked to not having a healthy lifestyle. You know, but you don't act e.g. with nutrition and sport.

The conclusion of the discussion can be summed up by another quote from a stakeholder:

The eight areas are very interlinked … lack of information is the core which starts the sequence of events.

Participants agreed that we had reached a stage of common understanding of the issues adolescents face in the area, and we were involved in jointly creating possible solutions to some of these issues.

### Establishing a stakeholder forum

We were able to convene 26 stakeholders representing 12 constituencies. Attrition was 50% overall, with 13 stakeholders in the third and final workshop. Table 2 shows the breakdown of stakeholders at each workshop by age and sex.

**Table 2:** Participants at each stage

Activiy	Total	Male	Female	Aged under 30 years	Aged 30 years and over
Workshop 1	26	9	16	11	14
Text message voting	26	9	16	11	14
Workshop 2	16	4	11	7	8
Workshop 3	13	7	6	6	7

Female participation was higher at all workshops except the final one. The ratio between younger and older participants was relatively even for all activities. Throughout the process of attending three workshops and voting via text message between workshops, stakeholders deliberated and actively engaged with each other and the researchers.

Attrition was high at 50% over the three workshops, with 35% (*n*=9) of participants coming to all three. Of the 31% (*n*=8) who came to two workshops, 15% (*n*=4) had genuine reasons for not attending the third, and 7% (*n*=2) sent a proxy to the third. For government officials, the main reason for not attending the second and third workshops was being called unexpectedly to meetings. For other community members, illness was a main reason for not attending the later workshops.

In the third workshop, stakeholders listed 14 organisations already working in the area that could be called upon by the stakeholder group and researchers to work with Project Ntshembo, ranging from eco-garden clubs in schools to LoveLife (www.lovelife.org.za), a non-governmental organisation that aims to reduce HIV in adolescents though promoting healthy lifestyles and self-esteem in youth.

The stakeholders agreed on a list of five criteria for inclusion in the Ntshembo Stakeholder Forum: having time to attend four meetings annually, being interested in working with adolescents, having the confidence to speak out in group settings, a diverse group of people, and commitment to the aims of Project Ntshembo. Participants chose 12 members for the stakeholder forum, all of whom had been involved in this process, with 8 of the 12 attending the third workshop.

## Discussion

This stakeholder involvement process provided a locally derived, empirical base for developing the intervention and allowed researchers to assess how aligned their objectives were with the views of the community in which the work was to take place. The adolescent health priorities generated were very important in the development of the intervention, as they pointed to a poor standard of health literacy, as well as the need for behavioural change techniques and theories to form an important part of the intervention development. Behaviour change is complex, and the intervention cannot focus solely on transfer of knowledge and skills, but needs to take into account adolescent perceptions of others, cultural and societal norms, adolescent and adult attitudes and beliefs about adolescent behaviours, and the degree to which the adolescent feels that he or she has the capacity and agency to change his or her behaviour (29).

Much discussion centred on peer pressure and the contribution that lack of social support makes to exacerbating the effects of peer pressure. Mention of the crossover and cascade effect reminded the researchers that there are interactions between many factors that affect health-promoting and health-seeking behaviours. We need to consider personal factors, such as self-efficacy and self-esteem, that might influence or be influenced by interpersonal factors such as peer pressure, how these fit within cultural and structural factors such as poverty, and whether the area is rural or urban (30).

There was not much discussion around HIV and its importance in adolescent health. This could be due to a number of reasons: perhaps the issue of HIV is too obvious to discuss, or there may still be denial of HIV as an important health issue in this age group. Alternatively, it could be that HIV is still stigmatised and it is difficult to talk about it in a group setting.

The stakeholder involvement process described in this paper demonstrates one method by which public engagement in health research can be achieved, at a collaborative level (31). Stakeholder involvement resulted in a diagram, developed through consensus, that showed how problems affecting adolescents’ health relate to each other. This diagram was used in the development of a viable intervention (29). The formation of a stakeholder forum, with stakeholders themselves setting the criteria for membership and voting for forum members, ensured that this first step in public engagement will lead to continued engagement throughout the planned intervention. The forum will work with the researchers throughout Project Ntshembo to further develop public engagement strategies, share preliminary findings, refine and target the interventions, discuss research progress, problem-solve, and identify policy champions to act on research findings. Ongoing engagement is one of the key principles of any engagement process (21).

Engagement is an inherently interactive activity, and for this reason the Delphi technique was adapted to include face-to-face discussion during workshops. This was an innovative way of obtaining agreement and facilitating involvement of all stakeholders. These discussions ensured stakeholders understood and actively engaged in the process.

We attribute the success of recruiting 26 stakeholders to the first workshop to the long-standing relationship with the community. Eight of the twelve elected Ntshembo Stakeholder Forum members were at all three workshops, and the other four had attended at least one workshop.

### Limitations

The exclusive use of the English language during all study activities may have been a barrier to free and full discussions, as English is not the vernacular in that area. It is also possible that the heterogeneity of the group might have affected participation of the lay stakeholders should they have felt that their views were not as relevant as the professional stakeholders. This could also have been true with younger stakeholders not feeling as secure about their opinions as the older stakeholders. It is possible that the face-to-face discussions may have influenced stakeholders’ voting choices.

## Conclusion

In order to address the problems facing adolescents in the Agincourt sub-district, we wanted to begin public engagement activities through stakeholder involvement with individuals from the community and public sector who had experience in and potential influence over the health and well-being of adolescents. The three workshops brought researchers and community members to a point where they agreed on the most important issues facing adolescents and developed a stakeholder forum within which to tackle these. Consensus was reached that the intervention needs to focus primarily on behavioural change to reduce peer pressure and to improve health literacy and health-seeking behaviours. When Project Ntshembo goes ahead, it will be important to have continuous support from the Ntshembo Stakeholder Forum, as community representatives. They need to be involved from the start-up phase of the project, where priority issues were discussed and agreed upon, through to the end of the project and beyond, when the impact of the intervention is evaluated and, if effective, potentially scaled up.

The rhetoric is that public engagement is important in health research, but in practice researchers may be uncertain as to how to conduct public engagement activities. If there is a clear aim, if the researchers and stakeholders understand why the engagement activities are necessary and if there is an expectation and understanding that the process will be dynamic, then stakeholder involvement has the potential for mutual benefit for both research and the community in which research is taking place.
